# Lymphatic filariasis control in Tanzania: infection, disease perceptions and drug uptake patterns in an endemic community after multiple rounds of mass drug administration

**DOI:** 10.1186/s13071-018-2999-x

**Published:** 2018-07-20

**Authors:** Yahya A. Derua, William N. Kisinza, Paul E. Simonsen

**Affiliations:** 1 National Institute for Medical Research, Amani Medical Research Centre, P.O. Box 81, Muheza, Tanzania; 2 Department of Veterinary Disease Biology, Faculty of Health and Medical Sciences, University of Copenhagen, Dyrlægevej 100, 1870 Frederiksberg C, Denmark

**Keywords:** Lymphatic filariasis, Microfilaraemia, Circulating filarial antigens, Hydrocele, Elephantiasis, Questionnaire, Tanzania

## Abstract

**Background:**

Lymphatic filariasis (LF) control in most countries of sub-Saharan Africa is based on annual mass drug administration (MDA) with a combination of ivermectin and albendazole, in order to interrupt transmission. However, attaining and maintaining high treatment coverage has been a challenge in many LF control programmes. This study was designed to elucidate reasons for continued transmission of LF in an endemic area of Tanga, northeastern Tanzania, where control activities based on MDA had been in place for eight years by the time of this study in 2012.

**Methods:**

A cross-sectional questionnaire survey was conducted in three sentinel villages used for monitoring the impact of MDA on LF transmission. A total of 747 individuals were interviewed, out of which 172 (23.0%), 27 (3.6%) and 49 (6.5%) had been shown to have circulating filarial antigens (CFA), microfilaraemia (MF) and LF gross lesions, respectively, prior to the interviews.

**Results:**

The interviewed population had a mean age of 33.7 years and a male to female ratio of 0.8. Males, individuals aged 30 years and above, peasants/fishermen and recent immigrants to the study communities were significantly more affected (CFA, MF and/ or LF gross lesions) than the other population groups. However, drug uptake rates were not significantly different between LF affected (those with CFA, MF and/ or LF gross lesions) and non-affected individuals. Likewise, drug uptake rates were not significantly different across different demographic parameters of the study population, some of which differed significantly in the level of infection. Moreover, it was found that misconceptions on how LF can be acquired were still evident, linking its transmission to witchcraft, heredity and sexual behaviour.

**Conclusions:**

The findings indicated that misconceptions about LF and its transmission still existed despite eight years of control activities in the area. Improved communication on the rationale of MDA and an enhanced drug delivery strategy that is adapted to the local settings and targeting important demographic groups that serve as reservoir of infection will help in reaching the elimination target within a reasonable timeframe.

## Background

Lymphatic filariasis (LF) is a major public health problem in many developing countries and one of the most prevalent of the neglected tropical diseases (NTDs) [1]. In sub-Saharan Africa, LF is caused by *Wuchereria bancrofti* and transmitted by *Anopheles* and *Culex* mosquitoes [2]. In this region, it has been estimated that more than 45 million people are affected by LF [3]. The disease has considerable mental and socio-economic consequences to the affected individuals, and has been ranked as a leading cause of long-term disability in the world [4]. LF is widespread in Tanzania where it has been estimated that nearly six million people live with debilitating manifestations of the disease [5].

LF was targeted for global elimination following a World Health Assembly resolution passed in 1997, and subsequently in 2000 the World Health Organization (WHO) launched the Global Programme to Eliminate Lymphatic Filariasis (GPELF) with a target of eliminating the disease by 2020 [6, 7]. The principal intervention measure of GPELF is interruption of transmission by annual mass drug administration (MDA) of a single dose of albendazole in combination with either diethylcarbamazine (DEC) or ivermectin to all eligible individuals in endemic areas [8]. The drug combination reduces the density of microfilariae in the blood and thereby the level of disease transmission in endemic areas. However, for this strategy to be effective, a high treatment coverage (estimated to range from 65–90%) successively for at least five to six years (corresponding to the life-span of the adult worms) is necessary [8, 9].

The MDA strategy has shown promise in LF transmission control by reducing onward transmission of the disease in many endemic countries [10–12]. Reports have shown that by using this strategy, some countries have managed to lower LF transmission indices to below the cut-off threshold set by the WHO for elimination [12–14]. However, transmission of LF still continues in some areas with ongoing MDA based control activities [10–12]. It has been predicted that endemic areas with high baseline prevalence and/ or presence of very efficient vectors will require relatively higher treatment coverage (at least 90%) to stop parasite transmission [9, 15]. In Ghana, 14 rounds of MDA did not stop the transmission of LF in districts with relatively high baseline prevalence, while control was possible in districts with low baseline prevalence [16]. Moreover, it has been suggested that continued transmission of LF in control programme areas may also be a result of lower than optimal drug uptake [11, 17]. Drug compliance has been found to be affected by several factors including fear of side effects, a general dislike of taking drugs, low motivation of drug distributors, lack of knowledge of the disease in question and inadequate communication on the rationale of MDA as previously summarized [18–20].

In Tanga district, northeastern Tanzania, seven rounds of annual MDA were completed between October 2004 and December 2011. During this period, irregularities were observed in the timing of MDAs (inter-MDA period longer or shorter than one year) and drug treatment coverages were generally on the lower side, as previously reported [11, 21, 22]. In this respect, several studies conducted in Tanzania have suggested the need for improved drug uptake in the affected communities for the target of LF elimination to be achieved [11, 17, 18]. As the deadline set for LF elimination is approaching, all factors that affect the effectiveness of the MDA strategy need to be elucidated such that programmatic adjustments can be made to achieve the target within a reasonable timeframe. The current study was designed to elucidate the possible reasons for the continued transmission of LF in an endemic area of Tanga, northeastern Tanzania, where control activities based on MDA had been in place for eight years by 2012.

## Methods

### Study sites

The study was conducted in three villages located in Tanga District in Tanga Region, northeastern Tanzania. The villages were originally selected as sentinel sites for monitoring the impact of MDA on LF infection and transmission, and detailed descriptions of the sites and findings have been reported elsewhere [11, 21–23]. In brief, the villages were Kirare (5°15'01"S, 39°01'40"E) located about 20 km south of Tanga city along the Tanga-Pangani road, Kiomoni (5°04'01"S, 39°03'17"E) located about 5 km northwest of Tanga city near the Tanga-Amboni road, and Kisimatui (5°11'0"S, 39°0'0"E) located about 17 km southwest of Tanga city along the Pongwe-Marungu road. A population census conducted by the study team in November 2011 recorded a total of 690, 504 and 651 individuals in the included parts of Kirare (Mashine and Mtambuuni hamlets), Kiomoni (Mabavu hamlet) and Kisimatui (Majengo hamlet), respectively [22].

### Study design

**Fig. 1:**
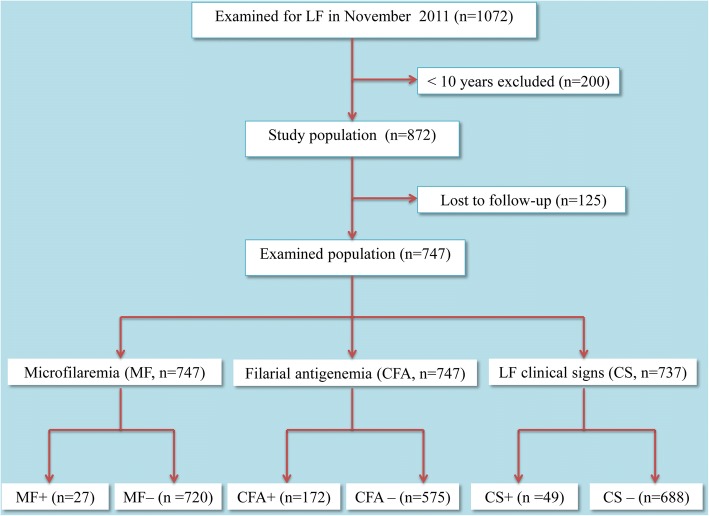
Study design

The current study was cross-sectional and questionnaire based. Individuals with known LF infection and chronic disease status as determined in a survey in November 2011 (immediately before the 7th round of MDA in December 2011) were interviewed in May 2012. In the 2011 survey, a total of 1072 individuals were examined for LF infection of which 42 had microfilariae (MF) and 213 had circulating filarial antigens (CFA) [22]. The methods used for diagnosing infections were described in detail previously [22], but briefly, MF were detected by counting chamber examination of 100 μl blood collected after 9 p.m., while CFA were detected in blood by use of immunochromatographic test cards (Binax Now Filariasis, Inverness Inc., Massachusetts, USA). Clinical examination moreover revealed that 54 of the individuals had gross chronic manifestations of LF (hydrocele and/or elephantiasis). Among the target population of 1072 individuals, 200 were excluded due to their young age (< 10 years) and 125 were lost to follow-up (Fig. 1). LF infection status in young school-children in the same sentinel villages has been reported previously [22, 23] and is generally low. Of 125 individuals who were lost to follow-up, 35 (28.0%), 6 (4.8%), 2 (1.6%) and 2 (1.6%) had CFA, MF, hydrocele and elephantiasis, respectively. Thus, the remaining 747 individuals from Kirare, Kisimatui and Kiomoni were included in the current study (Fig. 1).

### Questionnaire survey

The questionnaire survey was conducted to assess and compare characteristics of the filarial affected and non-affected individuals, especially on their perception of LF disease and control, and their reasons for taking or not taking the drugs during the MDAs. The questionnaire was prepared in English, translated into Kiswahili, pilot-tested in the field and revised accordingly before the actual data collection. Following the interviews, questionnaires were back-translated to English during data entry. The questionnaire surveys were carried out in May 2012 by trained field staff with experience in LF surveillance. By using a prepared list of participants (with serial number, hamlet number, house number, sex and age), interviewers visited their households and requested them to participate in the interviews. Although participants were known, interviewers were unaware of the infection and disease status of the individuals being interviewed (a blinded interview) except for those with obviously visible lesions. Interviewers visited a particular household at least twice to make sure that as many as possible of the individuals in the list were interviewed.

### Data analysis

Data collected were entered in an Excel database and transferred to SPSS (SPSS Inc., Chicago, IL, USA) for analysis. For the analyses, respondents were categorized in two age groups: 10–29 and ≥ 30 years. The level of education was categorized into two groups as either “none to not completed standard 7” or “at least completed standard 7”. A household wealth index was calculated based on availability of the following in a particular household: a mobile phone (1 point), a radio (2 points), a bicycle (3 points), a television (4 points), a refrigerator (5 points), a cow (6 points) and a motorcycle (7 points). The sum of points were calculated and categorized into zero (low income), 1–10 (medium income) and > 10 (high income). Different variables were compared using a Chi-square test and a *P*-value ≤ 0.05 was considered statistically significant.

## Results

A total of 747 individuals were interviewed from Kirare (37.9%), Kisimatui (32.7%) and Kiomoni (29.5%) villages. The male to female ratio was 0.8 and the mean age of the respondents was 33.7 years (range of 10–95 years). Of the interviewed individuals, 172 (23.0%), 27 (3.6%) and 49 (6.5%) had CFA, MF and LF gross lesions (hydrocele and/or elephantiasis), respectively (Fig. 1). Of the 49 respondents with LF gross lesions, 17 had elephantiasis with 4 (23.5%) and 1 (5.9%) having CFA and MF, respectively. Likewise, of the 49 respondents with LF gross lesions, 33 had hydrocele with 9 (27.3%) and 2 (6.1%) having CFA and MF, respectively. One individual had both hydrocele and elephantiasis. LF infection status among the respondents varied considerably by demographic characteristics of the study population (Table 1). Males and individuals aged 30 years and above were significantly more affected (CFA, MF and/ or LF gross lesions) than females and those below 30 years. With respect to occupation and duration of stay in the study villages, peasants/fishermen and recent immigrants (at most 10 years stay in the villages) were more affected than those engaged in other economic activities and natives (Table 1).

**Table 1:** Characteristics of the study population and their reported drug uptake in December 2011, in relation to their LF status (analyzed by Chi-square tests)

Characteristic	No. (% of total)	No. (%) with LF infection^a^	*χ*^2^-value (*P*-value)	No. (%) with LF mf^b^	*χ*^2^-value (*P*-value)	No. (%) with LF lesions^c^	*χ*^2^-value (*P*-value)	No. (%) with LF infection, mf and/or lesions	*χ*^2^-value (*P*-value)
Gender (*n* = 747)									
Female	422 (56.5)	78 (18.5)	11.290 (0.001)	10 (2.4)	4.314 (0.038)	12 (2.8)	23.018 (< 0.001)	88 (20.9)	23.599 (< 0.001)
Male	325 (43.5)	94 (28.9)		17 (5.2)		38 (11.7)		120 (36.9)	
Age group (*n* = 747)									
10–29 years	375 (50.2)	70 (18.7)	8.072 (0.004)	9 (2.4)	3.188 (0.074)	2 (0.5)	45.754 (< 0.001)	72 (19.2)	28.009 (< 0.001)
≥ 30 years	372 (49.8)	102 (27.4)		18 (4.8)		48 (12.9)		136 (36.6)	
School education (*n* = 747)									
None or not completed Std. 7	251 (33.6)	47 (18.7)	3.944 (0.047)	9 (3.6)	0.001 (0.976)	21 (8.4)	1.694 (0.190)	62 (24.7)	1.859 (0.173)
At least completed Std. 7	496 (66.4)	125 (25.2)		18 (3.6)		29 (5.8)		146 (29.4)	
Occupation (*n* = 747)									
Pupil/student	255 (34.1)	41 (16.1)	11.807 (0.003)	4 (1.6)	6.894 (0.032)	1 (0.4)	31.552 (< 0.001)	42 (16.5)	28.997 (< 0.001)
Peasant/fisherman	300 (40.2)	85 (28.3)		11 (3.7)		37 (12.3)		111 (37.0)	
Other^d^	192 (25.7)	46 (24.0)		12 (6.3)		12 (6.3)		55 (28.6)	
Stay in village (*n* = 746)^e^									
≥ 10 years	366 (49.1)	72 (19.7)	4.639 (0.031)	7 (1.9)	6.000 (0.014)	14 (3.8)	9.513 (0.002)	85 (23.2)	7.753 (0.005)
< 10 years	380 (50.9)	100 (26.3)		20 (5.3)		36 (9.5)		123 (32.4)	
Wealth index (*n* = 747)									
Low income	74 (9.9)	20 (27.0)	1.567 (0.561)	6 (8.1)	5.334 (0.069)	8 (10.8)	3.508 (0.173)	25 (33.8)	1.591 (0.451)
Medium income	500 (66.9)	116 (23.2)		14 (2.8)		28 (5.6)		134 (26.8)	
High income	173 (23.2)	36 (20.8)		7 (4.0)		14 (8.1)		49 (28.3)	
Reported drug uptake (*n* = 737)^f^									
Yes	573 (77.7)	129 (22.5)	0.444 (0.505)	19 (3.3)	0.882 (0.350)	38 (6.6)	0.001 (0.973)	159 (27.7)	0.052 (0.819)
No	164 (22.3)	41 (25.0)		8 (4.9)		11 (6.7)		47 (28.7)	

^a^CFA-positive

^b^Microfilariae-positive

^c^Hydrocele and/or elephantiasis

^d^Public/private employee, self-employed, housewife or no occupation

^e^One respondent did not answer/remember

^f^Ten respondents did not answer/remember

Most of the respondents (94.7%, *n* = 698) were aware of the ongoing MDA for LF control, but only 573 (77.7%) participated by swallowing the drugs in the December 2011 MDA campaign. Drug uptake rates were not significantly different between LF affected (those with CFA, MF and/ or LF gross lesions) and non-affected individuals (Tables 1, 2). Moreover, drug uptake rates were not different across different demographic parameters of the study population (Table 2).

**Table 2:** Reported drug uptake in December 2011 in relation to the demographic characteristics and LF status of the study population (analyzed by Chi-square tests)

Characteristic	No. (% of total)	No. (%) reported drug uptake	*χ*^2^-value	*P*-value
Gender (*n* = 737)				
Female	416 (56.4)	319 (76.7)	0.626	0.429
Male	321 (43.6)	254 (79.1)		
Age group (*n* = 737)				
10–29 years	370 (50.2)	277 (74.9)	3.569	0.059
≥ 30 years	367 (49.8)	296 (80.7)		
School education (*n* = 737)				
None or not completed Std. 7	246 (33.4)	183 (74.4)	2.406	0.121
At least completed Std. 7	491 (66.6)	390 (79.4)		
Occupation (*n* = 737)				
Pupil/student	251 (34.1)	191 (76.1)	2.270	0.321
Peasant/fisherman	298 (40.4)	240 (80.5)		
Other^a^	188 (25.5)	142 (75.5)		
Stay in village (*n* = 736)^b^				
≥ 10 years	362 (49.2)	291 (80.4)	2.931	0.087
< 10 years	374 (50.8)	281 (75.1)		
Wealth index (*n* = 737)				
Low income	72 (9.8)	55 (76.4)	4.455	0.108
Medium income	494 (67.0)	375 (75.9)		
High income	171 (23.2)	143 (83.6)		
LF infection^c^ (*n* = 737)				
Yes	170 (23.1)	129 (75.9)	0.444	0.505
No	567 (76.9)	444 (78.3)		
LF mf^d^ (*n* = 737)				
Yes	27 (3.7)	19 (70.3)	0.882	0.348
No	710 (96.3)	554 (78.0)		
LF lesion^e^ (*n* = 737)				
Yes	49 (6.6)	38 (77.6)	0.001	0.973
No	688 (93.4)	535 (77.8)		
LF infection, mf and/or lesion (*n* = 737)				
Yes	206 (28.0)	159 (77.2)	0.052	0.819
No	531 (72.0)	414 (78.0)		

^a^Public/private employee, self-employed, housewife or no occupation

^b^One respondent did not answer/remember

^c^CFA positive

^d^Microfilariae positive

^e^Hydrocele and/or elephantiasis

For the 573 individuals who took the drugs, the major reason for the uptake as reported by 469 (81.8%) respondents was to prevent them from getting LF. On the other hand, of 164 (22.3%) individuals who reported not to have taken the drugs, 75 (45.7%) had been absent at the time of drug distribution (Table 3).

**Table 3:** Reasons given for taking or not taking drugs in the December 2011 MDA campaign

Question/answer	No. of respondents (%)
Did you take the tablets in the 2011 MDA campaign? (*n* = 737)	
Yes	573 (77.7)
No	164 (22.3)
Why did you take the tablets? (*n* = 573)	
Prevent getting LF	469 (81.8)
Abiding leaders’ instructions	56 (9.8)
Other benefits of the drugs	12 (2.1)
Drugs given free of charge	6 (1.0)
Other people taking the drugs	3 (0.5)
No reason given	27 (4.7)
Why you did not take the tablets? (*n* = 164)	
Absent during the distribution	75 (45.7)
Drugs not distributed	8 (4.9)
Drugs contraindicated to me	9 (5.5)
Not informed about distribution time	27 (16.5)
Dislike the tablets	2 (1.2)
Afraid of side effects	4 (2.4)
I don’t have LF (tablets are for those with LF)	19 (11.6)
No reason given	20 (12.2)

The majority of the respondents reported that drugs were distributed at designated central places in the villages (59.7%, *n* = 423) by community health workers or members (69.3%, *n* = 475). Seventy-three percent (*n* = 537) of the respondents considered the methods deployed in the drug distribution to be convenient (Table 4). Of those who were not happy with the distribution method, some were of the view that drugs should be given by doctors (*n* = 3) and delivered at health facilities (*n* = 13). The reported drug uptake was significantly higher when the drugs were delivered to people’s homes/schools (Table 4). The reported drug uptake was not significantly different between different categories of distributors, although there was a trend of an increase in reported uptake when drugs distribution was conducted by health facility staff or village leaders.

**Table 4:** Responses to questions about drug distribution and perceived community health problems, and their relation to the reported drug uptake (analyzed by Chi-square tests)

Question/response	No. (% of total)	No. (%) reported drug uptake	*χ*^2^-value	*P*-value
Where were the drugs given? (*n* = 708)^a^				
From a central place	423 (59.7)	327 (77.3)	20.681	< 0.001
Brought home/school	175 (24.7)	161 (92.0)		
From the health facility	110 (15.5)	81 (73.6)		
Who distributed the drugs? (*n* = 685)^b^				
Community health workers/members	475 (69.3)	378 (79.6)	1.603	0.449
Health facility staff	123 (18.0)	101 (82.1)		
Village leaders	87 (12.7)	74 (85.1)		
Was the distribution method good? (*n* = 577)^c^				
Yes	537 (93.1)	478 (89.0)	2.947	0.086
No	40 (6.9)	32 (80.0)		
What do you consider most important health problem in your community? (*n* = 657)^d^				
Malaria	446 (67.9)	355 (79.6)	4.025	0.134
HIV/AIDS	123 (18.7)	91 (74.0)		
LF	88 (13.4)	75 (85.2)		
Do you consider LF a health problem in your community? (*n* = 699)^e^				
Yes	652 (93.3)	506 (77.6)	0.026	0.872
No	47 (6.7)	36 (76.6)		

^a^29 repondents who reported other minor distribution channels were excluded

^b^52 respondents who reported that other individuals distributed the drugs were excluded

^c^141 respondents who reported that they did not know and 19 who did not give any answer were excluded

^d^80 respondents were excluded [59 who reported health problems including pneumonia (*n* = 27), gastro-intestinal disorders (*n* = 17), skin infections (*n* = 6), flu (*n* = 4), schistosomiasis (*n* = 3), tuberculosis (*n* = 1) and high blood pressure (*n* = 1), in addition to 21 who replied that they did not know]

^e^38 respondents who replied that they did not know were excluded

Most of the respondents (93.3%, *n* = 652) were of the view that LF is a health problem in the study villages. However, the reported drug uptake between those who considered LF a health problem and those of the view that LF was not a health problem was not significantly different (Table 4). When asked to mention important disease conditions in their villages, LF ranked the third in the list after malaria and HIV/AIDS (Table 4). The reported drug uptake rates were not significantly different between the respondents who cited malaria, HIV/AIDS or LF as the most important disease condition (Table 4). Despite years of control activities based on MDA, LF was still poorly understood by the inhabitants in the study villages. More than half (53.3–56.0%) of the respondents were not aware how LF was transmitted. Furthermore, misconceptions were still evident on how LF can be acquired, linking its transmission to witchcraft, heredity and sexual behaviour (Table 5).

**Table 5:** Responses to questions about individuals’ perception of the mode of acquiring LF infection and lesions (*n* = 747)

Response	No. (% of total)		
	Mode of acquiring LF infection	Mode of acquiring hydrocele	Mode of acquiring elephantiasis
Don’t know	418 (56.0)	417 (55.8)	398 (53.3)
Mosquito bite	295 (39.5)	280 (37.5)	312 (41.8)
Stepping on unclean matter^a^	9 (1.2)	13 (1.7)	13 (1.7)
Living/sleeping with infected person	7 (0.9)	7 (0.9)	5 (0.7)
Sex with infected person	4 (0.5)	4 (0.5)	3 (0.4)
Sex during menstrual period	2 (0.3)	10 (1.3)	4 (0.5)
Witchcraft	2 (0.3)	2 (0.3)	6 (0.8)
Injury	2 (0.3)	2 (0.3)	2 (0.3)
Infection with parasites	3 (0.4)	2 (0.3)	–
Inherited	3 (0.4)	2 (0.3)	2 (0.3)
God’s decision	–	–	2 (0.3)
Other causes	2^b^ (1.9)	8^c^(1.1)	–

^a^Related to witchcraft

^b^Weather conditions (*n* = 2)

^c^High ambient temperature (*n* = 1), drinking coconut milk (*n* = 1), chronic hernia (*n* = 6)

## Discussion

Successful elimination of LF based on the MDA strategy relies on maintaining a high treatment coverage to reduce the worm burden in humans and hence the onwards transmission [9, 15]. However, attaining and maintaining a high treatment coverage has been a challenge in many LF control programmes globally, including the Tanzanian National Lymphatic Filariasis Elimination Programme [11, 17, 24–28]. Moreover, as the LF control campaigns progresses, the repeated rounds of MDA will eventually lead to fatigue among both programme implementers and those swallowing the drugs. It is crucial that all factors that affect effectiveness of the MDA strategy are understood and addressed to improve and maintain a high treatment coverage to accelerate the LF elimination efforts. With the deadline set to achieve global elimination of LF quickly approaching, renewed advocacy on MDA strategies adapted to local endemic communities needs to be intensified rather than relaxed.

The findings of the present study showed that reported drug uptake rates were not significantly different among those affected (with CFA, MF and/ or LF gross lesions) and non-affected. Moreover, the reported drug uptake rates were not significantly different across different demographic parameters of the study population, some of which were reservoirs of infection. Significantly more affection (CFA, MF and/ or LF gross lesions) was recorded in males, adults (≥ 30 years), peasants/fishermen and recently immigrated individuals (at most 10 years stay in the village). Studies conducted elsewhere have shown that the adult population, particularly males and peasants/fishermen, are disproportionally more likely to be infected and are hence important for the transmission of LF [29–31]. The reasons for high infection rates detected among the recent immigrants are not entirely clear, but we suggest that many of these individuals may have arrived from areas with no active MDA and/or may have been more exposed to infectious bites. MDA non-compliance among infected individuals (with reservoirs of worms) is of great concern as failure to treat these means that transmission continues. In this study, people with LF gross lesions were not found to be very important for ongoing LF transmission as relatively few (6.0%) were harbouring MF. Other studies have also reported high rates of amicrofilaraemia among patients with LF gross lesions [32, 33]. Importantly, it has also been shown elsewhere that implementation of morbidity management programs can have a very positive effect on MDA compliance [34].

In the villages of the present study, drugs were mainly offered at a designated central place, and distributed by community health workers. The majority of the respondents considered the mode of drug distribution to be convenient and appropriate. However, several individuals who were not comfortable with the distribution plan were of the view that drugs should be given by doctors or in health facilities. This point of view from a minority in the population needs to be considered, to improve drug uptake. Recruiting respected drug distributors empowered with adequate training and supervision was found to improved drug uptake rates elsewhere [35, 36]. On the other hand, major reasons reported by those who did not take the drugs were consequent to inadequate communication as to why people should swallow the drugs and inappropriate delivery time when some of the community members were not available in their respective villages (during school vacations and crop growing/harvesting seasons). These findings corroborate with those of others who have analyzed factors that cause lower than optimal treatment coverage in MDA programmes in different endemic countries [17, 18, 26, 37].

The findings of the present study have shown that even after several years of control activities, LF is still not well understood by nearly half of the people living in endemic communities, and its importance as a disease condition ranked well below that of malaria and HIV. Even individuals with gross lesions were not fully aware of the disease etiology, and did not consider it an important disease condition. Moreover, hydrocele and elephantiasis were still considered different entities by some of the respondents. Our findings on community perceptions on LF corroborate with those of others who have reported a variety of misconceptions surrounding LF in endemic communities neighbouring our study sites and elsewhere [18, 38]. It is encouraging that only a few individuals still considered LF a non-important disease. However, much still has to be done to educate the local communities on LF so they feel encouraged to participate effectively in elimination efforts.

Although reported drug uptake rates in the current study were relatively high (77.7%) and above the level recommended by the World Health Organization (65% or higher) [39], our findings suggest the need for adjustments in the MDA delivery. It is undisputable that a high treatment coverage is a predictor of successful LF elimination. However, our findings indicated that the population of LF parasite carriers in the community were treated at the same rate as those who were not infected. Thus continued delivery of MDA with this strategy will miss a good proportion of infected individuals and hence increase the duration necessary to reach the elimination target. As done for interventions targeting other diseases, innovation in delivering MDA is a requirement especially at the period when fatigue is expected, due to repeated rounds of drug uptake. We therefore suggest an enhanced MDA strategy and delivery mechanism that specifically targets adult males, peasants/fishermen and recent immigrants. To have a reasonable participation of the peasantry community, drugs should be distributed after the harvesting season. Drug distribution points in the fish markets will improve uptake in the fishermen community. Efforts should also be made to locate and treat recent immigrants who may not yet have proper housing or live in farms away from the village centre. In addition to improved communication, MDA needs to be tailored with events that will attract attention of the adult male population like sports, and MDA should be delivered by individuals who are adequately trained, motivated and respected by the community members.

## Conclusions

The findings of this study suggest that misconceptions surrounding LF disease and its transmission still exist despite eight years of control activities. Improved communication on the rationale of MDA and an enhanced drug delivery strategy that is adapted to the local settings and targeting important groups that serve as reservoir of infection will help in reaching the elimination target within a reasonable timeframe.

## Abbreviations

LF: lymphatic filariasis
MDA: mass drug administration
CFA: circulating filarial antigens
MF: microfilaraemia
NTDs: neglected tropical diseases
WHO: World Health Organization
GPELF: Global Programme to Eliminate Lymphatic Filariasis
DEC: diethylcarbamazine citrate
